# The Emerging Role of Tetraspanins in the Proteolytic Processing of the Amyloid Precursor Protein

**DOI:** 10.3389/fnmol.2016.00149

**Published:** 2016-12-21

**Authors:** Lisa Seipold, Paul Saftig

**Affiliations:** Institut für Biochemie, Christian-Albrechts-Universität zu Kiel (CAU)Kiel, Germany

**Keywords:** tetraspanin, Alzheimer disease, membrane microdomains, amyloid precursor protein, secretases, amyloid beta

## Abstract

Tetraspanins are a family of ubiquitously expressed and conserved proteins, which are characterized by four transmembrane domains and the formation of a short and a large extracellular loop (LEL). Through interaction with other tetraspanins and transmembrane proteins such as growth factors, receptors and integrins, tetraspanins build a wide ranging and membrane spanning protein network. Such tetraspanin-enriched microdomains (TEMs) contribute to the formation and stability of functional signaling complexes involved in cell activation, adhesion, motility, differentiation, and malignancy. There is increasing evidence showing that the tetraspanins also regulate the proteolysis of the amyloid precursor protein (APP) by physically interacting with the APP secretases. CD9, CD63, CD81, Tspan12, Tspan15 are among the tetraspanins involved in the intracellular transport and in the stabilization of the gamma secretase complex or ADAM10 as the major APP alpha secretase. They also directly regulate, most likely in concert with other tetraspanins, the proteolytic function of these membrane embedded enzymes. Despite the knowledge about the interaction of tetraspanins with the secretases not much is known about their physiological role, their importance in Alzheimer's Disease and their exact mode of action. This review aims to summarize the current knowledge and open questions regarding the biology of tetraspanins and the understanding how these proteins interact with APP processing pathways. Ultimately, it will be of interest if tetraspanins are suitable targets for future therapeutical approaches.

## Introduction

The neurotoxic amyloid beta (Aβ) peptide is a major component of senile plaques in Alzheimer's Disease (AD) and derives from its precursor the amyloid precursor protein (APP). Despite an intensive effort and increasing understanding of its role in AD the physiological function of APP is not completely understood. APP and its relatives amyloid-like protein-1 (APLP1) and amyloid-like protein-2 (APLP2) are proteolytically processed, ubiquitously expressed and share overlapping functions. APP has been linked with trophic roles in neurons and synapses, axon pruning, intracellular signaling and apoptosis (Muller and Zheng, 2012). How APP interaction with other proteins is defined, how its proteolytic processing is controlled and how signaling events are regulated by APP is poorly understood. Proteomics-based approaches and yeast-two-hybrid screens have been used to identify the protein interaction network of APP (Kohli et al., 2012; Yu et al., 2015) and of the proteases known to cleave APP (Wakabayashi et al., 2009; Jeon et al., 2013). Among others, members of the tetraspanin family have been identified. Tetraspanins have been characterized as scaffold for protein interactions establishing tetraspanin-enriched microdomains (TEMs) and are involved in grouping APP and functional important protein partners.

This review focuses on the emerging role of tetraspanins in the regulation of the proteases involved in the proteolytic processing of APP. The available knowledge about how tetraspanins regulate processing and intracellular trafficking of APP and APP-cleaving secretases is summarized. It is discussed why tetraspanins are attractive novel drug targets. There are some excellent reviews covering different aspects of tetraspanin biology thereby providing a useful overview about their diverse functions (Berditchevski and Odintsova, 2007; Yanez-Mo et al., 2009; Charrin et al., 2014).

## What are tetraspanins?

Tetraspanins are compact and glycosylated transmembrane proteins, that span cell membranes four times. Two extracellular domains, one larger and one smaller loop are separated from three cytosolic domains, one short loop and one N-terminal and C-terminal end, respectively. Intracellular cysteine residues of the tetraspanins can be modified by lipidation, i.e., addition of palmitate, possibly contributing to the establishment of tetraspanin microdomains and the regulation of intracellular signaling events (Berditchevski et al., 2002; Charrin et al., 2002; Yang et al., 2002). The large extracellular loop (LEL) and the transmembrane domains play a role in mediating protein-protein interactions (Hemler, 2003; Charrin et al., 2009). The structure of the isolated LEL of human CD81 was solved. It looks mushroom-shaped and it consists of a conserved subdomain, including three helices and a more variable one with two helices, possibly involved in the binding to other membrane proteins (Kitadokoro et al., 2001; Seigneuret et al., 2001). The full CD81 structure revealed a cone-like structure, where the LEL harbors an intramembrane cavity which is supposed to bind cholesterol (Zimmerman et al., 2016). It is speculated that the cholesterol bound structure favors a closed structural state of this tetraspanin with less tightly bound partner proteins.

Thirty three members of tetraspanins have been described. They can be mainly found at the plasma membrane and within endocytic membranes. Co-immunoprecipitation and crosslinking experiments revealed a high affinity of tetraspanins to interact with each other and other transmembrane proteins. These are in particular integrins, but also members of the immunoglobulin superfamily, signaling receptors, enzymes such as proteases and many other integral proteins residing in TEMs (Yanez-Mo et al., 2009).

## Functions of tetraspanins

The function of tetraspanins is mainly defined by their ability to interact with other transmembrane proteins. Due to the great variety of partner proteins, tetraspanins are involved in various cellular processes like migration, adhesion, signaling and pathogen infection (Boucheix and Rubinstein, 2001; Lammerding et al., 2003; Barreiro et al., 2008). By regulating cell motility and different signaling pathways, tetraspanins play an important role in cancer progression and metastasis (Boucheix and Rubinstein, 2001; Wang et al., 2011). For example, the tetraspanins CD9 and CD151 contribute to cancer cell invasion by interacting with different integrins and signaling enzymes, like protein kinase C (PKC) and phosphoinositide 4-kinase (PI4K) (Zhang et al., 2001; Wang et al., 2011). Tetraspanins also modulate intracellular signaling pathways by coordinating ligand-receptor binding at the cell surface. This is exemplified by the observation that tetraspanin 3 promotes binding of the NogoA ligand to the receptor sphingosine-1-phosphate-receptor-2 (S1PR2), which activates an intracellular signaling cascade leading to the inhibition of neurite outgrowth (Thiede-Stan et al., 2015). Most tetraspanins regulate the functions of their partner proteins by modulating their spatiotemporal distribution at the plasma membrane and organizing them together with other functional proteins (e.g., enzymes and substrates) (Odintsova et al., 2003; Haining et al., 2012; Thiede-Stan et al., 2015). Recent studies, demonstrated that the interaction with tetraspanins influences the motility of their partner proteins and their association with other molecules within the plasma membrane (Yang et al., 2012; Mattila et al., 2013; Jouannet et al., 2015). In addition, some tetraspanins directly control the trafficking of their partner proteins (Berditchevski and Odintsova, 2007). For example CD63, facilitates endocytosis of the HIV receptor C-X-C chemokine receptor type 4 (Yoshida et al., 2008).

Further genetic and *in vivo* studies demonstrated the importance of tetraspanins in various physiological and pathophysiological processes. In the central nervous system, the knockout of CD81 increased brain size and number of glial cells in mice (Geisert et al., 2002). Tspan7 regulates spine maturation and AMPA receptor trafficking by interacting with the protein interacting with C-kinase 1 (PICK1) in rat hippocampal neurons (Bassani et al., 2012). Moreover, loss of CD9 in mice impaired formation of axoglial paranodal junctions and caused myelination deficits in the peripheral nervous system (Ishibashi et al., 2004). Also other tetraspanins like Tspan5 (Garcia-Frigola et al., 2001) and Tspan3 (Seipold et al., 2016) are highly expressed in the brain and in neuronal cells. However, their physiological roles remain unclear. Tetraspanin knockout mice additionally revealed the importance of CD9, CD81, CD37, and CD151 for fertilization, brain and peripheral nerve development and the immune response. However, analysis of tetraspanin functions by loss-of-function approaches in mice has been hampered, due to compensatory effects and their redundant functions.

In human, mutations of tetraspanin 7, CD151 and the retinal tetraspanin Peripherin/RDS are associated with X-linked mental retardation, skin and kidney diseases, deafness and retinal degeneration (Kohl et al., 1998; Zemni et al., 2000; Karamatic Crew et al., 2004).

## Tetraspanins as regulators of α-secretase activity

Several tetraspanins associate with the APP secretases and regulate their activity. In particular, the membrane localized α-secretase ADAM10 associates with multiple tetraspanins. Using mild detergent conditions CD9, CD53, CD81, CD82, and CD151 were identified to associate with ADAM10. CD9, CD81, CD82 were able to stimulate ADAM10-dependent TNFα and EGF shedding (Arduise et al., 2008). In an independent study the association of ADAM10 with tetraspanin 12 (Tspan12) caused an accelerated ADAM10 maturation, i.e., the cleavage of the pro-ADAM10 to the mature and active protease, followed by an increased ADAM10-dependent APP processing (Xu et al., 2009). It was postulated that Tspan12 activated proprotein convertases and stabilized the mature form of ADAM10. Co-immunoprecipitation experiments, performed under stringent detergent conditions, suggested that CD9, CD81, CD82, and CD151 did not directly interact with ADAM10 (Dornier et al., 2012). It was concluded that these tetraspanins associate with ADAM10 through interactions mediated by other members of the tetraspanin web. However, tetraspanins belonging to the TspanC8 subfamily still interacted with ADAM10 under stringent immunoprecipitation conditions, indicating that these tetraspanins directly bind to the protease (Dornier et al., 2012).

**Figure 1:**
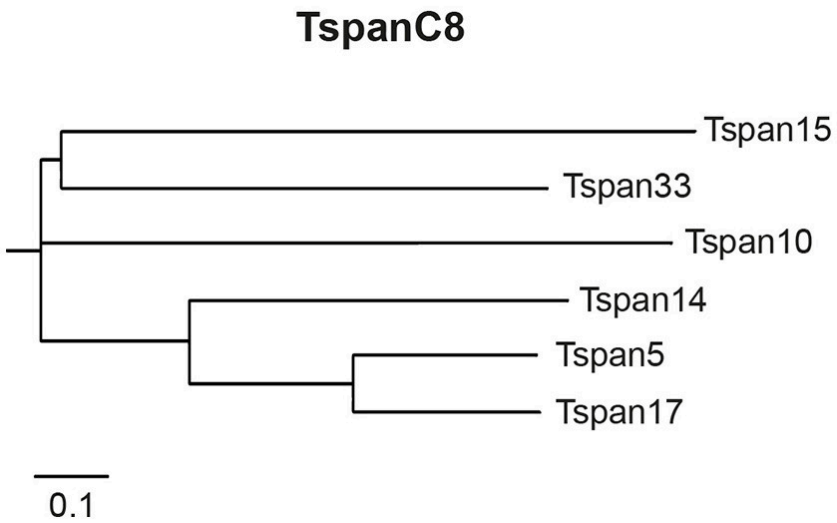
**The subgroup of TspanC8 tetraspanins**. The TspanC8 tetraspanins are an evolutionary conserved subgroup of tetraspanins, including Tspan5, Tspan10, Tspan14, Tspan15, Tspan17, and Tspan33. TspanC8 tetraspanins interact with the APP α-secretase ADAM10 and regulate its maturation, surface expression and substrate cleavage. Alignment of the human TspanC8 amino acid sequences was performed with ClustalOmega and is presented as dendrogram.

This evolutionary related subgroup of TspanC8 tetraspanins (Figure 1) includes the tetraspanins 5, 10, 14, 15, 17, and 33, which all contain eight conserved cysteine residues within their LEL. Analysis of the TspanC8-ADAM10 interaction revealed that overexpression of individual TspanC8 tetraspanins promoted ADAM10 maturation in human cells and *Drosophila melanogaster* (Haining et al., 2012). With exception of Tspan10 and Tspan17, TspanC8 overexpression also increased ADAM10 surface localization. Heterologous Tspan10 and Tspan17 expression led to a localization of ADAM10 to late endosomes (Dornier et al., 2012). Although, the C8 tetraspanins exert similar effects on ADAM10 maturation and trafficking (except Tspan10 and Tspan17), they have different impact on the cleavage of ADAM10 substrates (Prox et al., 2012; Noy et al., 2016). The overexpression of the TspanC8s members Tspan5 and Tspan14 promoted ligand induced shedding of the Notch receptor. In contrast, expression of Tspan15 reduced Notch processing (Dornier et al., 2012). Tspan15 was the only TspanC8 member, that increased ADAM10-mediated N-cadherin shedding after overexpression in human embryonic kidney (HEK293) and monkey fibroblast-like Cos7 cells (Prox et al., 2012; Noy et al., 2016). It was also shown that the generation of APP C-terminal fragments was differentially affected by certain TspanC8s. While expression of Tspan14 and Tspan33 slightly reduced the appearance of APP C-terminal cleavage products (Jouannet et al., 2015), Tspan5 expression had no effect on the production these fragments. Tspan15 expression in the human osteosarcoma cell line U2OS-N1 (Jouannet et al., 2015) also reduced APP processing, but increased it in murine neuroblastoma (N2a) and HEK293 cells (Prox et al., 2012). Since, tetraspanins act in concert with other tetraspanins in TEMs the conflicting data may be explained by the different composition of tetraspanins in the different cellular systems used.

There is increasing evidence that TspanC8s mediate substrate specificity by a direct interaction with ADAM10 and modulation of its association with other membrane components, e.g., integrins (Jouannet et al., 2016). Using ADAM10 chimeric and truncation constructs, it was demonstrated that the TspanC8s differentially favor the interaction with the ADAM10 membrane proximal stalk region, cysteine-rich domain and disintegrin domain (Noy et al., 2016). TspanC8s may constrain and stabilize ADAM10 in defined conformations (Noy et al., 2016). The expression of TspanC8 tetraspanins had different impact on the membrane environment of ADAM10. Mass-spectrometric analysis of ADAM10-associated proteins revealed that in Tspan5 expressing cells ADAM10 preferably associated with classical components of the tetraspanin web such as the α3β1 integrin, CD9P1 and CD9, which was reduced in Tspan15 transfected cells (Jouannet et al., 2015). Tspan5 expression enhanced ADAM10's localization at the cell periphery, while Tspan15 expression did not (Jouannet et al., 2015).

Tspan3, a TspanC6 tetraspanin, was identified, as another ADAM10 and APP interacting tetraspanin in cells and in the murine brain (Seipold et al., 2016). Tspan3 expression did not obviously influence ADAM10 maturation or trafficking but increased ADAM10-mediated APP cleavage. Tspan3 is likely involved in this process as a scaffold protein, which stabilizes ADAM10 and APP at the cell surface.

An interaction of tetraspanins with ADAM17, which is closely related to ADAM10 and under certain circumstances also exerts α-secretase activity toward APP (Buxbaum et al., 1998), has only been described for CD9. Heterologous expression of CD9 or treatment with CD9-specific antibodies inhibited phorbol ester (PMA)-stimulated shedding of the ADAM17 substrates TNFα and ICAM-I, while CD9 knockdown increased it (Gutierrez-Lopez et al., 2011). In the same manner, treatment with neutralizing anti-CD9 monoclonal antibodies reduced ADAM17-mediated shedding of LR11 (SorLa) in human leukocytes, while CD9 expression increased it (Tsukamoto et al., 2014). Most likely CD9 inhibits ADAM17 sheddase activity by affecting the membrane compartmentalization of ADAM17 itself or its substrates. CD9 may even interact with both, ADAM10 and ADAM17, but exerting opposite effects on their activity with regards to TNFα shedding (Gutierrez-Lopez et al., 2011).

## Tetraspanins as regulators of γ-secretase activity

Next to ADAM10 also the γ-secretase complex interacts with tetraspanins. Wakabayashi et al. showed that Presenilin-1 and Presenilin-2 associate with the tetraspanins CD9, CD81, Upk1b as well as with the tetraspanin associated proteins EWI-F, EWI-2 which connect the tetraspanin web with the actin cytoskeleton (Sala-Valdes et al., 2006) and CD98hc (Fenczik et al., 2001), a regulator of integrin signaling and amino acid transport (Wakabayashi et al., 2009). The activity of the γ-secretase complex was strongly decreased upon knockdown of CD81, EWI-F, and CD98hc, which correlated with a decrease in Aβ production. Inhibition of γ-secretase activity was also observed in CD9 and CD81-deficient mouse embryonic fibroblasts revealed by an accumulation of C-terminal fragments of the γ-secretase substrates APP, APLP-2 ADAM10, N-Cadherin and Syndecan-3. Treatment with anti-CD9 monoclonal antibodies reduced Aβ levels in HEK293 cells (Wakabayashi et al., 2009). Additionally, siRNA mediated knockdown of Tspan33 in HeLa cells reduced the γ-secretase dependent cleavage of a constitutively active, truncated form of Notch1 and that of T-cell acute lymphoblastic leukemia (T-ALL) Notch1 oncogenic mutants (Dunn et al., 2010).

Independent studies analyzing the interactome of the γ-secretase complex identified the tetraspanins CD63 and Tspan3 in the network of the presenilin interacting proteins (Jeon et al., 2013; Seipold et al., 2016). CD63 is one of the few tetraspanins which is found on late endosomal and lysosomal membranes (Rous et al., 2002). CD63 associates with CD9, CD81, and CD82 within the tetraspanin web. However, the functional consequence of this interaction for the γ-secretase complex has not been elucidated. Due to its functions in the endosomal sorting complex required for transport (ESCRT)-independent formation of intraluminal vesicles (van Niel et al., 2011) CD63 could control the degradation of the γ-secretase complex.

## Tetraspanins as a part of a multi-secretase complex

**Figure 2:**
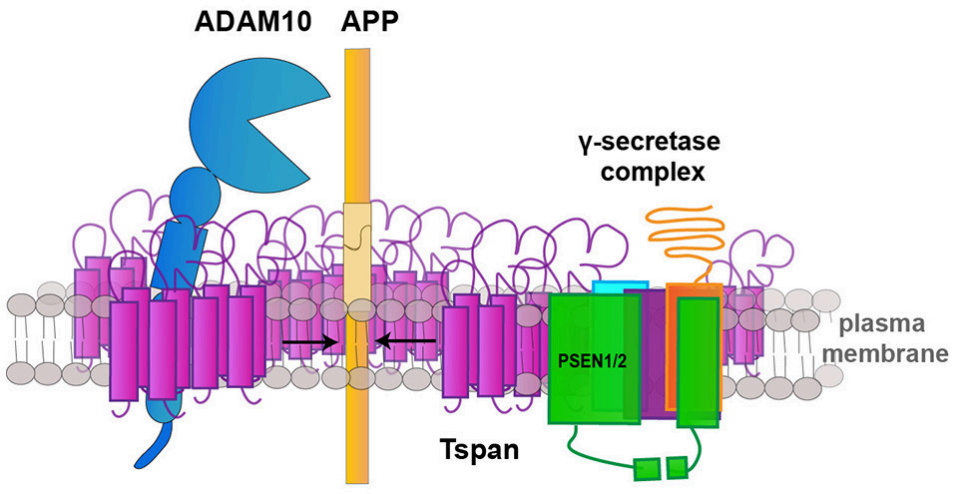
**Tetraspanins regulate APP cleaving enzymes**. Schematic drawing illustrating the role of tetraspanins (Tspan) as scaffolds for the assembly of a multisecretase complex, which consists of ADAM10 and the γ-secretase-complex and is required for the processing of the amyloid precursor protein (APP) at the plasma membrane.

Another mechanism by which tetraspanins regulate both, α- and γ-secretase, activity has recently been reported by Chen et al. (2015). It was shown that α- and γ-secretase associate in an active multiprotease complex at the plasma membrane (Figure 2). Assembly of this multi-secretase complex seems to be modified by Tspan12 and the TspanC8 tetraspanins Tspan 5, 14, 12, and 17. Knockdown of Tspan5 and Tspan14 decreased ADAM10 association with the γ-secretase complex, which correlated with a reduced presence of mature ADAM10. Knockdown of Tspan12 and Tspan17 also decreased the association of ADAM10 with the γ-secretase complex and the α-secretase-dependent generation of soluble sAPPα, but did not alter ADAM10 maturation. Moreover, Tspan12 and Tspan17 seem to contribute to an α-/γ-secretase feedback mechanism. This feedback mechanism is related to γ-secretase inhibition and causes an increase of sAPPα at the expense of sAPPβ. This is also accompanied by an increase of APP and BACE1 surface levels. This effect was less effective after knockdown of Tspan12 and Tspan17 (Chen et al., 2015).

In conclusion, tetraspanins are potent regulators of α- and γ-secretase activity, which modulate maturation, complex assembly, trafficking and substrate specificity. In regards to β-secretase cleavage, no direct interaction of tetraspanins with the β-secretase BACE1 has been reported. The metalloprotease Meprin β, which cleaves APP in a manner similar to BACE1 (Bien et al., 2012), interacts with Tspan8 and resides together with APP in TEMs (Schmidt et al., 2016). However, Tspan8 had no impact on the proteolytic activity of Meprin β towards APP.

## Tetraspanins as therapeutic targets

The treatment with monoclonal antibodies (mAbs) against CD81 diminished the development of neurological symptoms in a multiple sclerosis mouse model (Dijkstra et al., 2008) and prevented hepatitis C virus (HCV) infection after prophylactic injection in mice (Meuleman et al., 2008). Application of anti-CD9 antibodies reduced tumor growth and progression in gastric cancer mouse xenografts (Nakamoto et al., 2009). Stimulatory CD151 antibodies promoted cell adhesion and thereby reduced immobilization of tumor cells and metastasis (Zijlstra et al., 2008). The humanized anti-CD37 IgG fusion protein Otlertuzumab (TRU-016) is a potential drug for the treatment of lymphoid B-cell malignancies (Robak et al., 2009) and was tested in phase 1 clinical trials for the treatment of chronic lymphocytic leukemia (Byrd et al., 2014). It is proposed that mAbs directed against tetraspanins inhibit lateral associations or cause the formation of tetraspanin aggregates, which disrupt TEMs and cause a downregulation of the targeted tetraspanin and its partner protein(s) (Hemler, 2008). Also recombinant soluble large extracellular domains (sLEL) may inhibit tetraspanin-dependent functions. Similar to mAbs, CD81 sLELs reduced HCV infectivity and blocked HIV-1 entry into macrophages (Flint et al., 2006; Ho et al., 2006).

The potential of tetraspanins to modulate γ-secretase activity in AD was demonstrated by RNAi-mediated knockdown experiments. The downregulation of CD81 and tetraspanin-associated proteins EWI-F and CD98hc reduced the secretion of neurotoxic Aβ in HEK cells, stably overexpressing a mutated form of APP, which favors amyloidogenic processing. Likewise, treatment with anti-CD81 and anti-CD9 mAbs decreased Aβ-production in HEK293 cells (Wakabayashi et al., 2009). However, most therapeutic approaches targeting γ-secretase activity were accompanied by severe side effects, like skin cancer development, gastrointestinal toxicity and infections (Doody et al., 2013), due to the physiological role of its substrates, for example Notch1 (Haapasalo and Kovacs, 2011). Interestingly, the individual knockdown of CD9 and CD81 in HeLa cells had no significant effect on the activity of different leukemic mutant forms of Notch1 (Dunn et al., 2010). It was further shown that human Tspan33 promotes γ-secretase cleavage of Notch and that depletion of Tspan33 might be a potential target in T-ALL, a rare yet aggressive form of lymphoblastic leukemia, which is associated with activating mutations of Notch1 (Weng et al., 2004; Dunn et al., 2010). Since CD9, CD81, and Tspan33 are also regarded as regulators of ADAM10- and ADAM17 (Arduise et al., 2008; Gutierrez-Lopez et al., 2011; Haining et al., 2012; Jouannet et al., 2016), the effects of potential therapeutics have to be studied carefully.

By reducing Aβ- and increasing sAPPα-production the upregulation of ADAM10 expression had beneficial effects in an AD mouse model (Postina et al., 2004). ADAM10 is another promising target for the treatment of AD, as demonstrated by a recent study using the synthetic retinoid acitretin to increase ADAM10 expression in AD patients (Endres et al., 2014). Targeting specific members of the TspanC8s, which enhance ADAM10 activity, but have different impact on its substrate specificity, could possibly reduce side effects of a global ADAM10 activation. Moreover, most of the TspanC8s are not expressed in all cell-types (Dornier et al., 2012; Jouannet et al., 2016), indicating that targeting these tetraspanins could regulate ADAM10 activity in a cell-type specific manner. With regard to AD, enhancing ADAM10 non-amyloidogenic APP processing could be achieved by stimulation of Tspan12, Tspan15 and Tspan33 using agonistic monoclonal antibodies, sLELs or small molecular drugs that increase the promoter activity and protein expression of these tetraspanins.

ADAM10 is also associated with tumor progression, metastasis and inflammation by site-specific cleavage of several adhesion molecules and cytokines. In this case a downregulation of its proteolytic activity could be of therapeutic benefit (Moss et al., 2008; Saftig and Reiss, 2011). ADAM10-mediated N-cadherin shedding was associated with cancer cell migration (Kohutek et al., 2009) promoting tumor progression and metastasis. In this regard downregulation of Tspan5 and Tspan15, which predominantly promote ADAM10-mediated N-cadherin shedding (Noy et al., 2016), by antagonistic mAbs, sLELs or RNAi, could be a therapeutic option. By sharing several substrates with ADAM10, inhibition of ADAM17 is also effective in different kinds of cancer and inflammatory disorders (Saftig and Reiss, 2011). The expression of CD9 reduced ADAM17-dependent TNFα shedding (Gutierrez-Lopez et al., 2011), which is a main factor in inflammation and involved in rheumatoid arthritis, psoriasis and inflammatory bowel disease.

To evaluate the full therapeutic potential of tetraspanins, the exact mechanisms and consequences of potential tetraspanin-directed therapeutics need to be further investigated. Due to their multiple interaction partners and the complex organization in TEMs, tetraspanins also have opposing functions, which might depend on the cellular system. While downregulation of Tspan33 in HeLa cells decreased Notch1 signaling (Dunn et al., 2010), its overexpression in U2OS-N1 cells reduced Notch1 activity (Jouannet et al., 2016). Targeting such tetraspanins could cause severe adverse effects such as cancer development and inflammation. Moreover, redundancy of tetraspanin functions and compensatory effects might decrease the clinical activity of potential therapeutics.

## Conclusion

In summary, tetraspanins are potent regulators of APP cleaving enzymes. In particular, tetraspanins came into focus as cell-type and substrate specific regulators of the α-secretases ADAM10 but also of the γ-secretase complex.

Their specific functions and localization make tetraspanins an interesting target for the treatment of AD and possibly other diseases. However, first approaches trying to target tetraspanins have not succeeded, which could be explained by their functional redundancy. It will be necessary to better understand how tetraspanins exactly work and how their redundancy is regulated. Another central aspect is how tetraspanin expression is regulated and if tetraspanin dysfunctions are associated with the development of AD.

## Author contributions

Both authors wrote the review manuscript. LS designed the figure art.

## Funding

Work in the laboratory of PS has been funded through grants of the Deutsche Forschungsgemeinschaft (DFG) in the SFB877, A3, and A12; through support of the VERUM foundation, the Alzheimer Research Price of the Breuer Foundation and the Interuniversity Attraction Poles Program IUAP P7/16 of the Belgian Federal Science Policy Office.

### Conflict of interest statement

The authors declare that the research was conducted in the absence of any commercial or financial relationships that could be construed as a potential conflict of interest.
