# Corrigendum: The Emerging Role of Tetraspanins in the Proteolytic Processing of the Amyloid Precursor Protein

**DOI:** 10.3389/fnmol.2017.00037

**Published:** 2017-02-10

**Authors:** Lisa Seipold, Paul Saftig

**Affiliations:** Institut für Biochemie, Christian-Albrechts Universität zu KielKiel, Germany

**Keywords:** tetraspanin, Alzheimer disease, membrane microdomains, amyloid precursor protein, secretases, amyloid beta

There was a mistake of a statement on page 5 (4th paragraph). It should read: “… In this regard downregulation of Tspan15, which predominantly promotes ADAM10-mediated N-cadherin shedding (Noy et al., 2016), by antagonistic mABs, sLELs, or RNAi, could be a therapeutic option….”. The authors apologize for the mistake.

## Conflict of interest statement

The authors declare that the research was conducted in the absence of any commercial or financial relationships that could be construed as a potential conflict of interest.

